# PDIA3 gene induces visceral hypersensitivity in rats with irritable bowel syndrome through the dendritic cell-mediated activation of T cells

**DOI:** 10.7717/peerj.2644

**Published:** 2016-11-17

**Authors:** Zhaomeng Zhuang, Lu Zhang, Xiaoteng Wang, Liyuan Tao, Bin Lv

**Affiliations:** 1 Zhejiang Chinese Medical University, Hanzhou, China; 2 Wenzhou Integrated Traditional Chinese and Western Medicine Hospital, Wenzhou Shi, Zhejiang Sheng, China

**Keywords:** IL-4, PDIA3, Dendritic cell, CD4+/CD8+ T lymphocyte, IL-9, Visceral hypersensitivity

## Abstract

This study investigated the mechanism of protein disulfide-isomerase A3 (PDIA3)-induced visceral hypersensitivity in irritable bowel syndrome (IBS). Rats were treated with saline (control), acetic acid and restraint stress (IBS model), empty vector (RNAi control) and PDIA3-RNAi vector (PDIA3-RNAi). Mesenteric lymph node DCs (MLNDCs) and splenic CD4+/CD8+ T cells were isolated for co-cultivation. Compared with control, MLNDCs co-cultured with CD4+ or CD8+ T cells showed an increased ability to promote T cell proliferation and produced more IL-4 or IL-9 secretion. Compared with the RNAi control, MLNDCs from the PDIA3 knockdown models were less effective in promoting the proliferation of CD4+/CD8+ T cells. It is concluded that PDIA3 plays an important role in the development of IBS through the DC-mediated activation of T cells, resulting in degranulation of MCs and visceral hypersensitivity.

## Introduction

Irritable bowel syndrome (IBS) is one of the most common functional gastrointestinal disorders characterized by the presence of abdominal pain or discomfort and associated with altered bowel habits, causing significant heathy issue globally (Spiegel, 2009). The causes and pathogenesis of IBS are still largely unclear. A growing number of studies suggest that immune dysregulation is closely related to the pathogenesis of IBS and mucosal immune dysfunction plays an important role in the pathogenesis of IBS. However, more details need to be elucidated.

In the mucosal immune system antigen-presenting cell (APC) plays a crucial role in activating T cells, and dendritic cells (DCs) are the major APCs (Cremon et al., 2009; Liebregts et al., 2007). Studies have shown that the infiltration and release of mast cell (MC) in colon are the cause of the symptom in IBS patients, and are closely related to visceral hypersensitivity in patients with IBS (Ohman & Simren, 2010). During the MC activation, cytokines IL-4 and -9 secreted by T cells play an important role (Bugajev et al., 2010; Kashiwada et al., 2010).

In our previous study, we found that the expression of PDIA3 (also called ERp57), a member of protein disulfide-isomerase family, is significantly upregulated in the colon mucosa tissues of IBS rats (Ding et al., 2010), suggesting that PDIA3 may be involved in IBS pathogenesis. Other studies showed that PDIA3 may be involved in antigen presentation process (Ghaith et al., 2010). Therefore, we speculated that PDIA3 may promote endogenous antigen presentation to increase the sensitivity and reactivity of DCs to the antigens, resulting in excessive immunity of T cells to overproduce IL-4 and IL-9, leading to the generation of highly sensitive MC, or even granulated MC, activated protease activating receptor-2 (PAR-2), and consequently visceral hypersensitivity in IBS (Zhuang et al., 2015).

In this study, we investigated the effects of PDIA3 on DC activation and visceral sensitivity using rat IBS models to provide better understanding of mechanism underlying IBS.

## Materials and Methods

### Animals, modelling and knockdown

Forty-eight healthy SD rats (250 ± 10 g) were purchased from the Animal Center of the Third Military Medical University, Chongqing. All rats arrived without deformity, trauma, or skin infections. The rats were raised in cages with pellet foods and water ad libitum, at 25 °C under 12 h lighting:12 h dark condition. Animal experiments were performed after 1 week of adaptive feeding. The experimental protocols were approved by the Zhejiang Chinese Medical University Ethics Committee. The approval number was SYXK (Zhejiang) 2013-0184.

### Visceral hypersensitivity model and PDIA3 knockdown

The rats were injected daily with 150 µL saline (*n* = 12), empty virus (RNAi control, *n* = 12) or PDIA3-RNAi lentiviruses (*n* = 12) (Western-biotechnology Co., Ltd., Chongqing) at a titer of 5 × 10^8^ TU through the tail veins for 3 days. Three days later, intracolonic instillation of 4% acetic acid combined with restraint stress was performed to construct visceral hypersensitivity models as described (La et al., 2003; Williams et al., 1988). Control rats were intracolonically instillated with saline or 150 µL saline following an intracolonic instillation of 4% acetic acid (model control). The hypersensitivity was assessed using abdominal withdrawal reflex (AWR) as described (Al-Chaer, Kawasaki & Pasricha, 2000).

### Sample collection

On day 2 following the acetic acid instillation, two rats in each group were randomly selected and anesthetized with 3% pentobarbital (40 mg/kg). Colon tissues (1 cm long) were collected 6 cm above the anus to evaluate acute intestinal mucous damage induced by acetic acid. On day 10 after acetic acid instillation, the remaining rats were anesthetized and decapitated. Peripheral blood (about 5 ml) was collected and centrifuged at 3,000 rpm for 10 min. The serum was stored at −80 °C until use. The ileocecal tissues and the colon tissues 6 cm above anus were isolated, longitudinally cut, cleaned with saline, and divided into two parts, which were either soaked in 10% neutral formalin or stored in liquid nitrogen.

### Western blot and ELISA

Protein extracts were prepared with a cell lysis reagent (Sigma-Aldrich, St. Louis, MO, USA) according to the manual, and the protein was quantified by a BCA assay (Pierce, Rockford, IL, USA). Then, the protein samples were separated by SDS-PAGE (10%) and detected by Western blot using polyclonal (rabbit) anti-IL-4, anti-IL-9 and anti-GAPDH (Abcam, USA) antibodies. Goat anti-rabbit IgG (Pierce, Rockford,IL, USA) secondary antibody conjugated to horseradish peroxidase and ECL detection systems (SuperSignal West Femto, Pierce) were used for detection. IL-4 and IL-9 levels were analyzed using ELISA kits for IL-4 (eBioscience, USA) and IL-9 (GUSABIO, USA) according to the manufacturer’s protocols.

### Dendritic cell isolation

DCs were isolated as reported (Fazal, 2013) using Anti-DC (OX62) Micreadeads (Miltenyi Biotech Inc., Auburn, CA) from the lymphoid tissues per instructions of the manufacturer.

### CD4+/CD8+ T cell: DC co-culture assays

CD4+/CD8+ T cells and DCs at (10:1 ratio) were co-cultured in 96-well flat-bottomed microtiter plates (Falcon, Lincoln Park, NJ, USA) for 7days at 37 °C and 5% CO_2_. Cells were harvested at the end of culture period and centrifuged to obtain the supernatant.

### Flow cytometry

FACS sorting was used to sort out DCs after labeling with fluorescent labels. Flow cytometry was also used for the determination of T cell type and activation cell markers during the experiments. DCs were labeled with respective FITC/PE/APC-tagged rat-specific antibodies at 1–5 µg/ml per 10^6^ cells. Isotype control antibodies and unstained cells were used as controls.

### Cell viability assay

Cell viability was evaluated using a tetrazolium-based MTT assay (CellTiter 96 AQueous One Solution Reagent; Promega, Madison, WI, USA). Briefly, cells were seeded in 96-well plates at a density of 2,500 cells per well, 20 µl MTS reagent was added to each well and the cells were incubated for one additional hour. The absorbance was measured at 490 nm using an automated microplate reader (Bio-Rad, Hercules, CA, USA). Each test was performed in triplicate.

### Statistical analysis

Data were analyzed using SPSS17.0 statistical software. The quantitative data were presented as means ± SD. Student’s *t*- test was performed to compare the means of two independent samples. Value with *P* < 0.05 was considered statistically significant.

## Results

### PDIA3 knockdown

**Figure 1:**
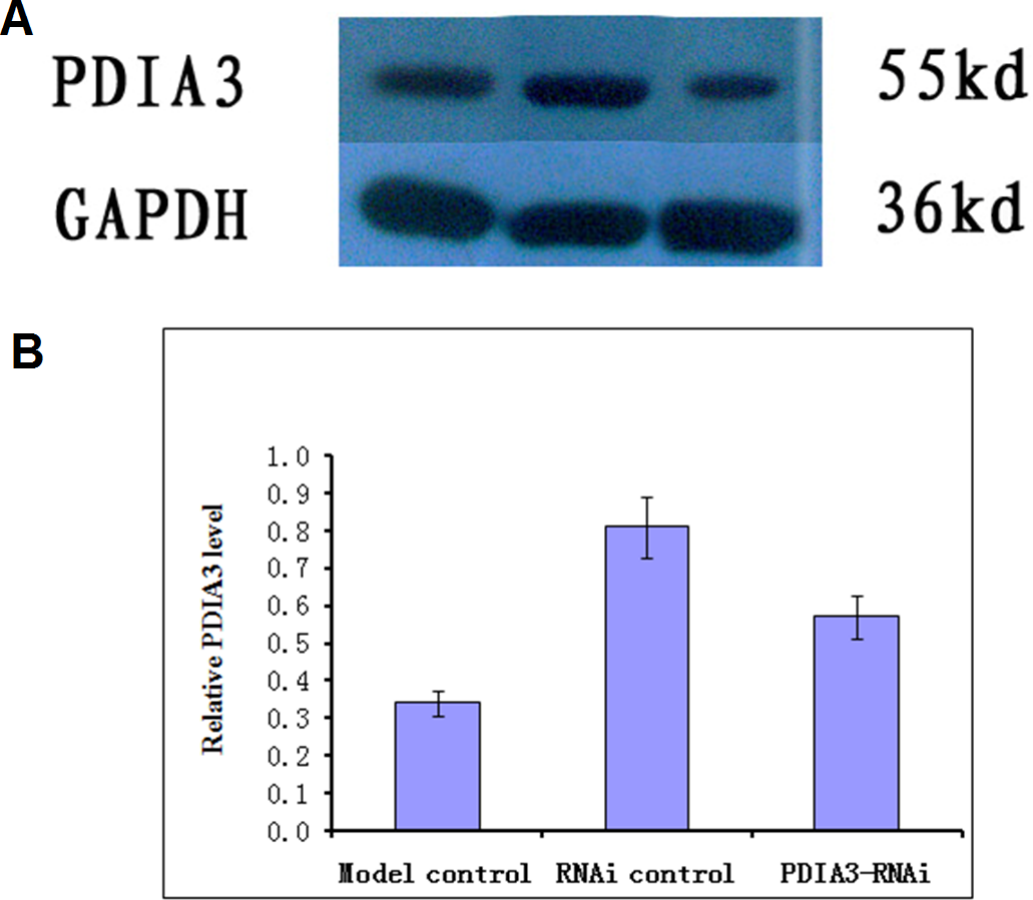
Western blot analysis of PDIA3 in IBS models after PDIA3 knockdown with RNAi. (A) Representative Western blots; (B) PDIA3 expression relative to GADPH. Control, rat models injected with saline solution; empty vector, rat models injected with empty vector; RNAi-PDIA3, rat models injected RNAi-PDIA3. All experiments were repeated three times, and difference in the PDIA3 expression levels were analyzed using *t*-test. ## denotes significant difference (*P* < 0.05) vs control or empty vector.

Virus without or with PDIA3- RNAi was injected into rats via the tail vein for three days, and the expression of the PDIA3 gene in the ileocecal tissues was detected with Western blot analysis. The results showed the relative expression levels were 0.57 ± 0.04 in control, 0.81 ± 0.03 and 0.34 ± 0.04 in RNAi control and RNAi model, respectively (Fig. 1). Statistical analysis showed that PDIA3 protein level was significantly (*P* < 0.05) lower in RNAi model than in control or RNAi control, indicating that *PDIA3* was successfully down-regulated.

### Animal models

PDIA3-RNAi or RNAi control rates exhibited normal behaviors with similar feeding and defecating as those in control. In the first 1–2 days after modeling, all rats exhibited restless, watery stool and perianal contamination with fecal residues, increased drinking, and decreased food consumption. On day 4–5, rats in the modeling groups defecated soft fece with less water. Starting from day 7 when the restraint stress was applied, all rats in modelling groups defecated mainly soft stools, occasionally particle-like feces. After the instillation of acetic acid and restraint stress were completed, the models appeared listlessness with shrugged hairs, reduced activity and slow response. The stools were thin and soft with perianal fecal residue. They significantly reduced water and food consumption.

### IBS model assessment

The visceral sensitivity was assessed based on defecation between 2 pm and 4 pm on day 10, and balloon pressure difference measured on day 10 at AWR = 3. The results showed that the sensitivity was significantly higher in model control or RNAi control than rats in control (*P* < 0.05), and lower in RNAi models (*P* < 0.05) (Table 1), whose sensitivity was similar to control (Table 1). Toluidine blue staining showed that MCs were mainly distributed in the loose connective tissue of the colon. The number of MCs in the model control was significantly higher than in the control (2.17 ± 0.72 vs 0.58 ± 0.51, *P* < 0.05), and significantly reduced in the RNAi models as compared to those in RNAi control (0.67 ± 0.65 vs 2.17 ± 0.72, *P* < 0.05). There was no significant difference between control and model control (*P* > 0.05). Under the electron microscope, MCs were intact with homogeneous cytoplasm in control or RNAi models. However, MCs showed broken cell membrane and released particles in model control and RNAi control.

**Table 1:** Defecation and balloon pressure of rats subjected to the instillation of acetic acid and restraint stress and injection of PDIA3-RNAi on day 10.

Rats	No. hard stools	No. soft stools	No. loose stools	Total no. stools	Balloon pressure (mmHg)
Control	7	2^**^	0	9	77.21 ± 7.35^**^
Model control	5	9^*^	1	15	50.25 ± 5.62^*^
RNAi control	5	5^**^	1	11	79.14 ± 8.06^**^
PDIA3-RNAi	8	11^*^	2	21	41.67 ± 5.62^*^

**Notes.**

* denotes *P* < 0.05 vs control.

** denotes *P* < 0.05 vs RNAi.

### CD103 expression

**Figure 2:**
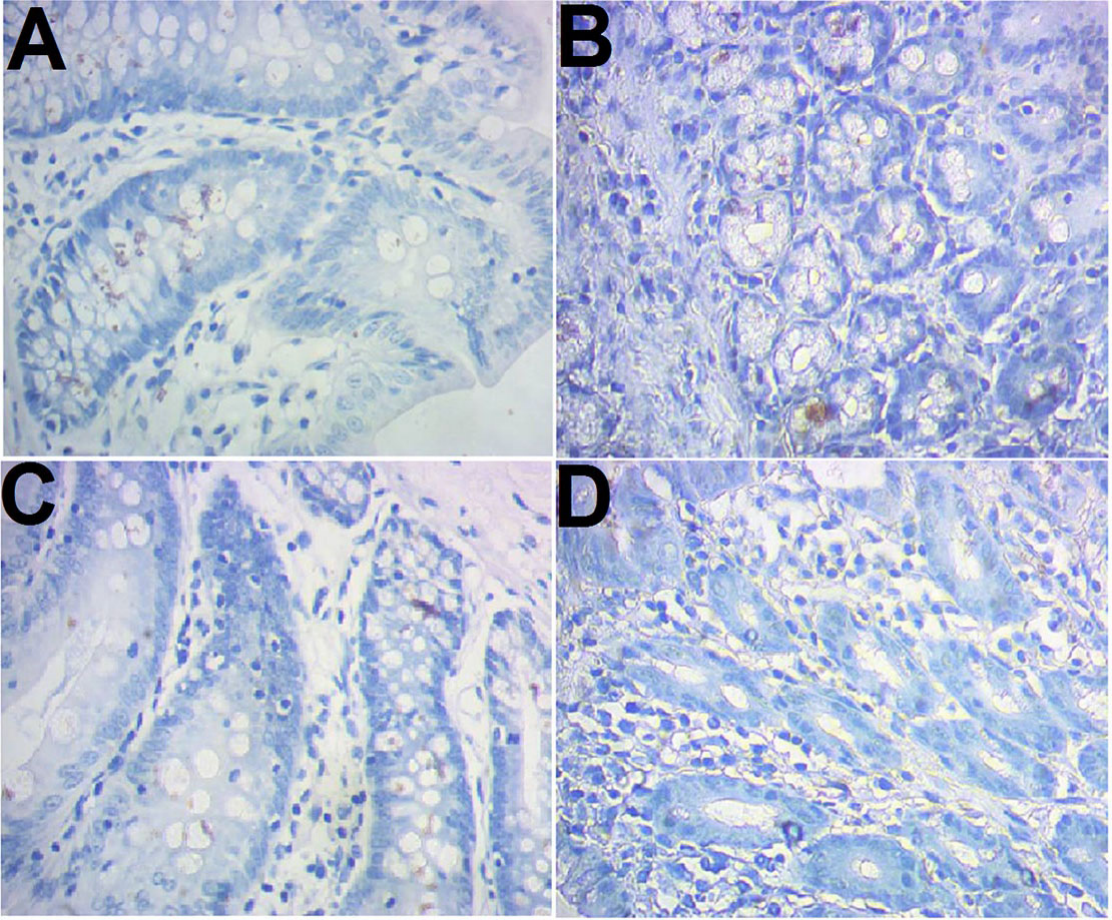
Immunohistochemical staining of CD103+ ileocecal DC cells. (A) Control (normal rat); (B), model control (rat models injected with saline solution); (C), RNAi control (rat models injected with empty vector); (D) RNAi model (rat models injected RNAi-PDIA). Ileocecal tissues were isolated after modelling, fixed in 10% neutral formalin and sectioned. The slides were incubated with CD103 antibody and examined after color development. Brown-colored cells were rated as positive for CD103.

CD103 expression was examined immunohistochemically. The results showed that there were more brown-colored CD103+ DCs in the RNAi models than control (10.83 ± 1.03 vs 6.25 ± 1.14, *P* < 0.05), less in RNAi control rats compared to the RNAi models (7.42 ± 0.90 vs 10.83 ± 1.03, *P* < 0.05). No significant difference was observed between the control and model control (Fig. 2).

### Promotion of DCs on proliferation of CD4+/CD8+ T cells

**Figure 3:**
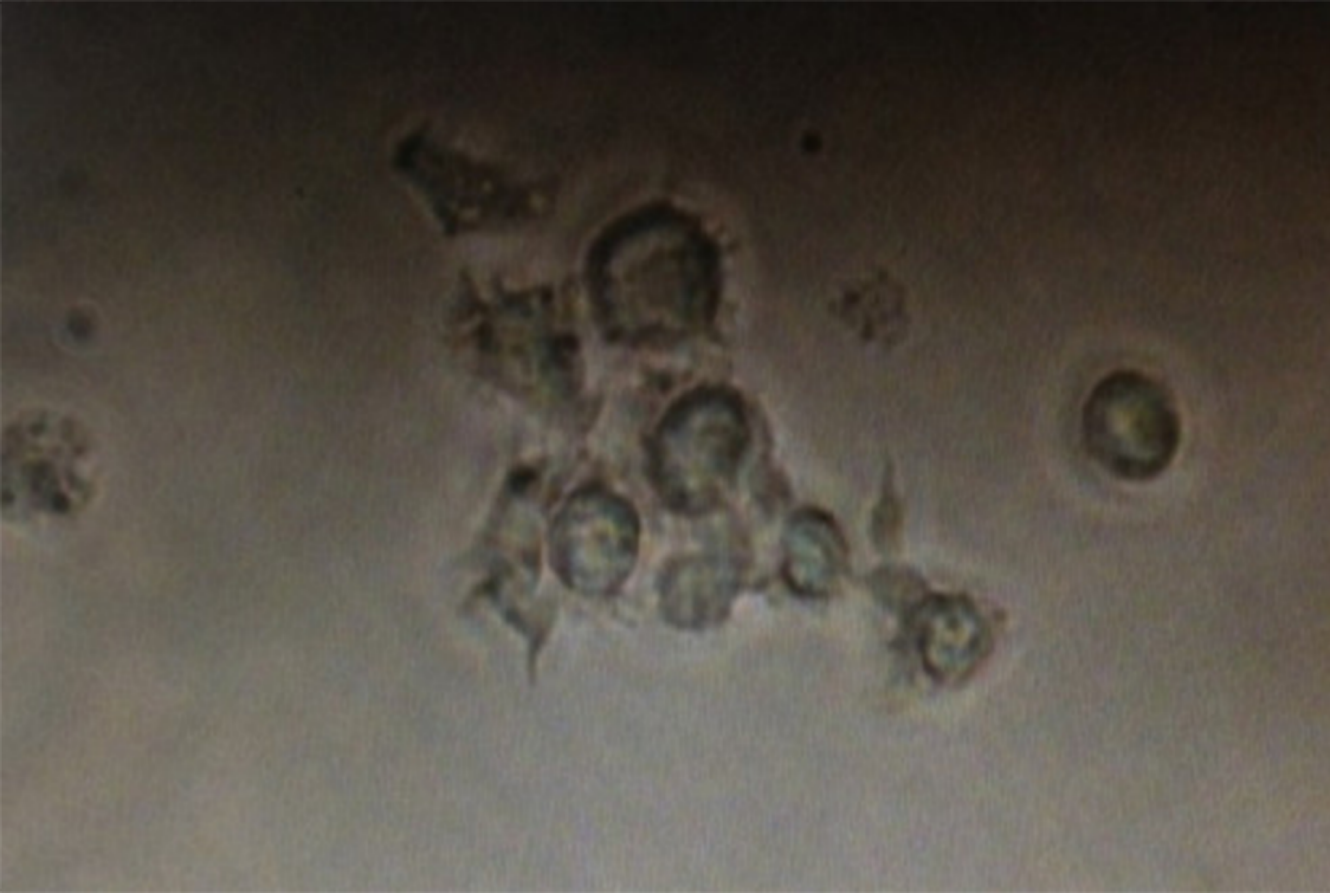
Dendritic cells acquired after flow sorting with typical dendritic protrusions on the cell surface. DCs were isolated using Anti-DC (OX62) microbeads from lymphoid tissues, purified by FACS sorting, and photographed.

Spleen T cells were isolated and flow cytometry showed that on average 97.66 ± 6.87% and 97.19 ± 7.32% of the cells were CD4+ or CD8+, respectively (S1-2); trypan blue staining showed that on average, the cell viability was >90% and 97.46 ± 1.87%, respectively. On average, 97.41 ±1.87% of freshly isolated MLNDCs were positive for PE (S3) and showed typical dendritic protrusions and on the cell surface when cultured *in vitro* (Fig. 3). The maturation of DCs were further examined using CD80 as makers and over 90% of the DCs were positive for CD80 when co-cultured with the T cells for three days, while none of them was positive if cultured without the T cells (S4). When co-cultured with CD4+/CD8+ T cells, the MLNDCs from RNAi control had stronger promotive effect on the T cell proliferation as compared with control (Table 2), while the promotion was reduced in RNAi rats compared to the RNAi control (Table 2).

**Table 2:** Proliferation and secretion of cytokines of CD4+/CD8+ T lymphocyte co-cultured with DC.

Source of DC	No. CD4+ T cell	No. CD8+ T cell	IL-4 from CD4+ T cell (pg/ml)	IL-4 from CD8+ T cell (pg/ml)	IL-9 from CD4+ T cell (pg/ml)	IL-9 from CD8+ T cell (pg/ml)
Control	0.54 ± 0.01	0.52 ± 0.01^**^	10.24 ± 0.09	7.35 ± 0.12	15.86 ± 10.19	29.12 ± 5.14
Model control	0.60 ± 0.01^*^	0.59 ± 0.00^*^	16.61 ± 1.00^*^	13.91 ± 0.57^*^	43.51 ± 11.32^*^	60.70 ± 11.02^*^
RNAi control	0.56 ± 0.01^*^	0.56 ± 0.01^*^	14.59 ± 0.80^*^	11.79 ± 0.42^*^	39.91 ± 9.73^*^	50.32 ± 6.95^*^
PDIA3-RNAi	0.53 ± 0.01^**^	0.54 ± 0.01^**^	11.75 ± 0.54^**^	8.63 ± 0.24^**^	29.05 ± 2.09^**^	37.17 ± 2.65^**^

**Notes.**

* denotes *P* < 0.05 vs control.

** denotes *P* < 0.05 vs RNAi control.

### Promotion of DCs on secretion of IL-4 and IL-9 in CD4+/CD8+ T cells

When CD4+/CD+ T cells were co-cultured with DCs from RNAi control, the secretion of IL-4 and IL-9 increased compared with the controls (Table 2, *P* < 0.05). On other hand, the cytokine secretions were reduced when CD4+/CD8+ T cells were co-cultured with DCs from RNAi models compared with the RNAi control. No significant difference was observed between the control and RNAi control (Table 2). Compared with co-cultivation with CD4+ T cells, co-cultivation with CD8+ T cells produced significantly less IL-9 (*P* < 0.05, Table 2).

## Discussion

Recent studies have found that mucosal immune dysfunction plays an important role in the pathogenesis of IBS, colonic mucosa of patients with IBS has increased number of immune cells (CD3+, CD4+ and CD8+ T cells) and increased production of cytokines (such as IL-5, IL-13, IL-6, TNF-*α*, and IL-1*β*) (Feili-Hariri, Falkner & Morel, 2005; Ohman & Simren, 2010; Vickery et al., 2011). In our early study, PDIA3 was found highly expressed in the colonic mucosa of IBS rats (Ding et al., 2010), as well as in patients with diarrhea IBS. Recent studies have found that PDIA3 plays an important role in endogenous antigen presentation. Antoniou et al. (2007) reported that PDIA3 molecules can directly insert into the peptide binding groove of the MHC class I molecules to bind with them during the course of MHC molecules antigen-presenting, making the antigen binding groove more bindable for antigen peptides (Santos et al., 2007; Stepensky, Bangia & Cresswell, 2007), resulting in increased binding stability between MHC molecules and antigen peptides.

Our experiments showed that the models had reduced balloon pressure, suggesting that their visceral sensitivity was increased, while PDIA3-RNAi models had reduced defecation and increased balloon pressure, suggesting that their visceral sensitivity is reduced. Since no difference in defecation was found between the controls, the reduction is likely due to PDIA3 knockdown. Furthermore, we found that in the model control and RNAi control, the serum and mucosal IL-4 and IL-9 and number of MCs were higher than in the model, with more degranulated MC and up-regulated TPS expression. On other hand, in PDIA3 knockdown models, these parameters were reduced. These are consistent with other works in IBS patients (Cenac et al., 2007; Weston et al., 1993).

The importance of MCs in IBS has been confirmed. After activation, MC releases mediators such as histamine, 5-serotonin and fibrinolytic enzyme to activate sensory neurons including those in the gastrointestinal tract. The fibrinolytic enzyme can activate PAR-2 located in the cell membrane of the spinal cord of the colon, causing sustained high excitation of PAR-2 (Cenac et al., 2007).

IL-4 is a cytokine that specifically prompts IgE generation. The up-regulation of IL-4 results in the production of antigen-specific IgE that activate MC (Kashiwada et al., 2010). IL-9 increases the expression of FceRI*α* on the surface of in MC, making MC more easily activated (Louahed et al., 1995). In earlier studies. There were reports showed that IL-4 was up-regulated in acute stage of mice with postinfectious irritable bowel syndrome, but restored to normal and even reduced (Akiho et al., 2005). IL-9 is usually considered as Th2 cytokine. However, in certain circumstances, a variety of T cells (Th9 cells, Th17 cells, TregCD8+ cells) can secrete IL-9 (Li et al., 2011), among them, MC is the main source (Wiener, Falus & Toth, 2004).

As a major professional antigen presenting cell, DC play an important role in innate and acquired immunity (Liebregts et al., 2007). DC is widely distributed in many tissues such as the intestinal lamina propria, Peyer’s patches and mesenteric lymph nodes in the intestinal mucosal immune system. DC located in the intestinal lamina propria and Peyer’s patches can uptake the antigens directly or by extending DCs into the intestine and migrating mesenteric lymph nodes for presenting to T cells to induce immune response. DC can provide necessary antigen peptide—MHC (first signal), CD80, CD86 and other costimulatory molecules (second signal) for T cell activation, induce naive T cells (Th0) differentiation to Th1 and Th2 and initiate T cell immune response to produce characteristic cytokines such as IL-2, IL-12, IFN-*γ* (Th1 pathway) and IL-4, IL-5, IL-9 (Th2 pathway), respectively (Feili-Hariri, Falkner & Morel, 2005). Therefore, we analyzed the number of ileocecal DCs before and after IBS modeling, and found that the number of the cells increased in the models, and the DCs had increased ability to promote the T cell proliferation with elevated cytokine secretion. However, little is known about the relationship between DC and IBS. Long et al. (2012) showed that after acute infection of lamblia, CD86 and MHC were expressed at low level in DC in mouse intestinal inherent layer and 8 week later, the expression was up-regulated. They found that at the acute stage, when DC was co-cultured with initial T cells, TH2 immune responses was produced, after PI-IBS stage, Th1 and Th17 immune response were produced, suggesting that there are DC-specific immune responses. In the PDIA3-RNAi models, however, the number of ileocecal DCs was reduced and these DCs were less effective in promoting T cell proliferation. These results suggest that DC may play an important role in the formation of visceral hypersensitivity in IBS by the activation of spleen CD4+/CD8+ to produce excessive IL-4 and IL-9 under local or systemic stress; knockdown of PDIA3 expression reduces the number and activity of intestinal DCs in visceral hypersensitivity rats, inhibits the abnormal increase of MC in serum and colon and the activity of abnormally secreted cytokines. This would block the up-regulation of trypsin to avoid prolonged high excitation PAR-2 in intestinal tissue, and reduce the visceral sensitivity. Therefore, we speculate that PDIA3 may increase the binding of MHC molecules synthesized in DCs to the antigens, leading to increased presenting of DCs to the antigens. This would subsequently promote the proliferation of autologous CD4+ and CD8+ T lymphocytes, stimulate the secretion of cytokines IL-4 and IL-9 to activate the downstream mast cells, resulting in the occurrence and development of visceral hypersensitivity.

Taken together, it is clear that the intestinal DCs may promote the spleen CD4+, CD8+ T cell activation to produce more IL-4 and IL-9, resulting in visceral hypersensitivity in IBS, while PDIA3 knockdown can block the process and reduce the sensitivity by reducing the number of intestinal DCs and DC-induced T cell proliferation in these processes. However, due the complexity of IBS, more works are needed to deliberate more specifically how PDIA3 increase the number of DC and activate DC, and whether the process is led by PDIA3.

## Conclusion

Under local or systemic stress, the proliferation of CD4+/CD8+ T cell and production of IL-4 and IL-9 are promoted by intestinal DC, resulting in high sensitivity or even degranulation of MC, leading to visceral hypersensitivity. Knockdown of the PDIA3 gene inhibits the abnormal immune response of CD4+/CD8+ T lymphocytes initiated by PDIA3 and mediated by DC, reducing the visceral sensitivity. PDIA3 promotes the occurrence of IBS visceral hypersensitivity through regulating the antigen presentation of DC to mediate an abnormal immune response of CD4+/CD8+ T lymphocytes and MC.

##  Supplemental Information

10.7717/peerj.2644/supp-1 Data S1 Balloon pressure measurements Original data for Table 1.

10.7717/peerj.2644/supp-2 Data S2 Cytokine measurements Original data for Table 2.

10.7717/peerj.2644/supp-3 Data S3 CD4 and CD8 positive T cells Original data for Table 3.

10.7717/peerj.2644/supp-4 Supplemental Information 1 Multicolor FACS analyses of freshly isolated T cells (A–D) Representative data from a multicolor assay of freshly isolated T cells stained for CD4 derived from control rats, Model-control, RNAi-control and PDIA3-RNAi. Green-colored subpopulation indicates CD4+ cell; each number indicates the percentage in the parent population.

10.7717/peerj.2644/supp-5 Supplemental Information 2 Multicolor FACS analyses of freshly isolated T cells (A–D) Representative data from a multicolor assay of freshly isolated T cells stained for CD4 derived from control rats, Model-control, RNAi-control and PDIA3-RNAi. Green-colored subpopulation indicates CD8+ cell; each number indicates the percentage in the parent population.

10.7717/peerj.2644/supp-6 Supplemental Information 3 Multicolor FACS analyses of freshly isolated DC cells (A–D) Representative data from a multicolor assay of freshly isolated DC cells stained for PE derived from control rats, Model-control, RNAi-control and PDIA3-RNAi. Green-colored subpopulation indicates CD8+ cell; each number indicates the percentage in the parent population.

10.7717/peerj.2644/supp-7 Supplemental Information 4 Multicolor FACS analyses of cultured DC cells (A) and (B) Representative data from a multicolor assay of DC cells stained for CD80 after three days of culture without (A) and with (B) T cells. Blue-colored subpopulation indicates CD80+ cell; each number indicates the percentage in the parent population.

## Additional Information and Declarations

### Data Availability

The following information was supplied regarding data availability: The raw data has been supplied as Datas S1–S3.
