# Supplementary figures and images for: PDIA3 gene induces visceral hypersensitivity in rats with irritable bowel syndrome through the dendritic cell-mediated activation of T cells
Source: PeerJ. 2016 Nov 17;4:e2644. doi: 10.7717/peerj.2644 (PMC5119228; doi:10.7717/peerj.2644)

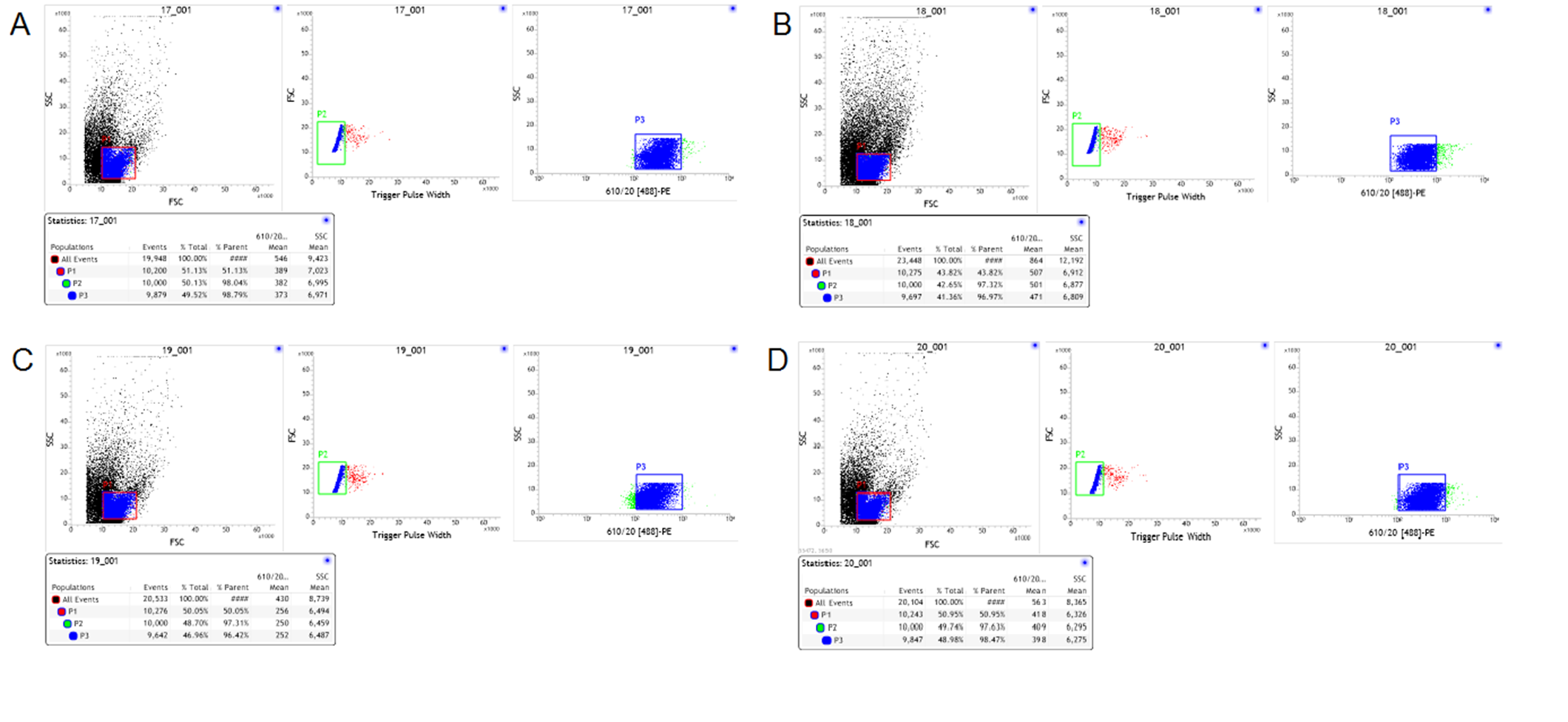

Supplement: Supplemental Information 1 — (A–D) Representative data from a multicolor assay of freshly isolated T cells stained for CD4 derived from control rats, Model-control, RNAi-control and PDIA3-RNAi. Green-colored subpopulation indicates CD4+ cell; each number indicates the percentage in the parent population. [file peerj-04-2644-s004.png]

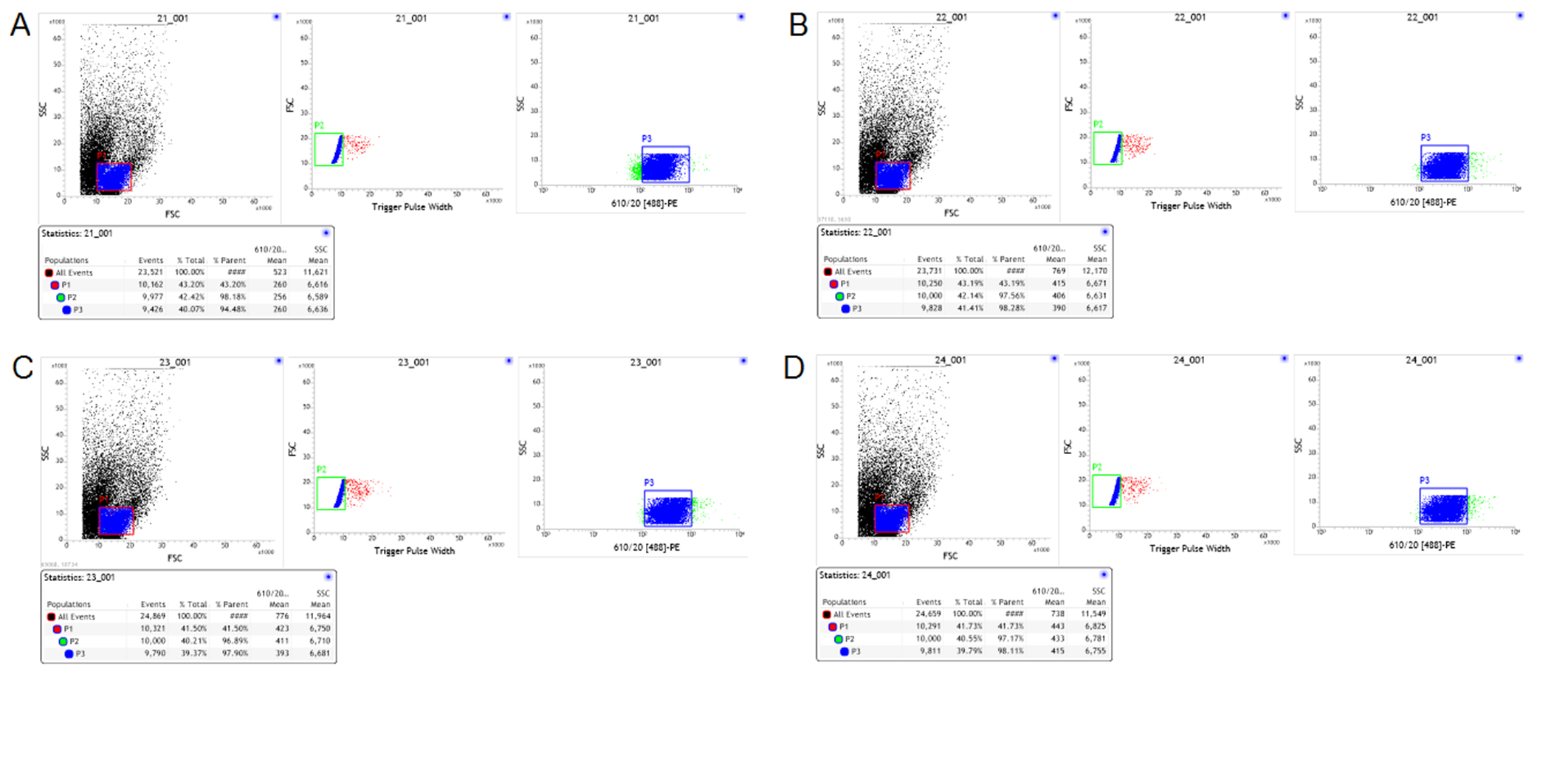

Supplement: Supplemental Information 2 — (A–D) Representative data from a multicolor assay of freshly isolated T cells stained for CD4 derived from control rats, Model-control, RNAi-control and PDIA3-RNAi. Green-colored subpopulation indicates CD8+ cell; each number indicates the percentage in the parent population. [file peerj-04-2644-s005.png]

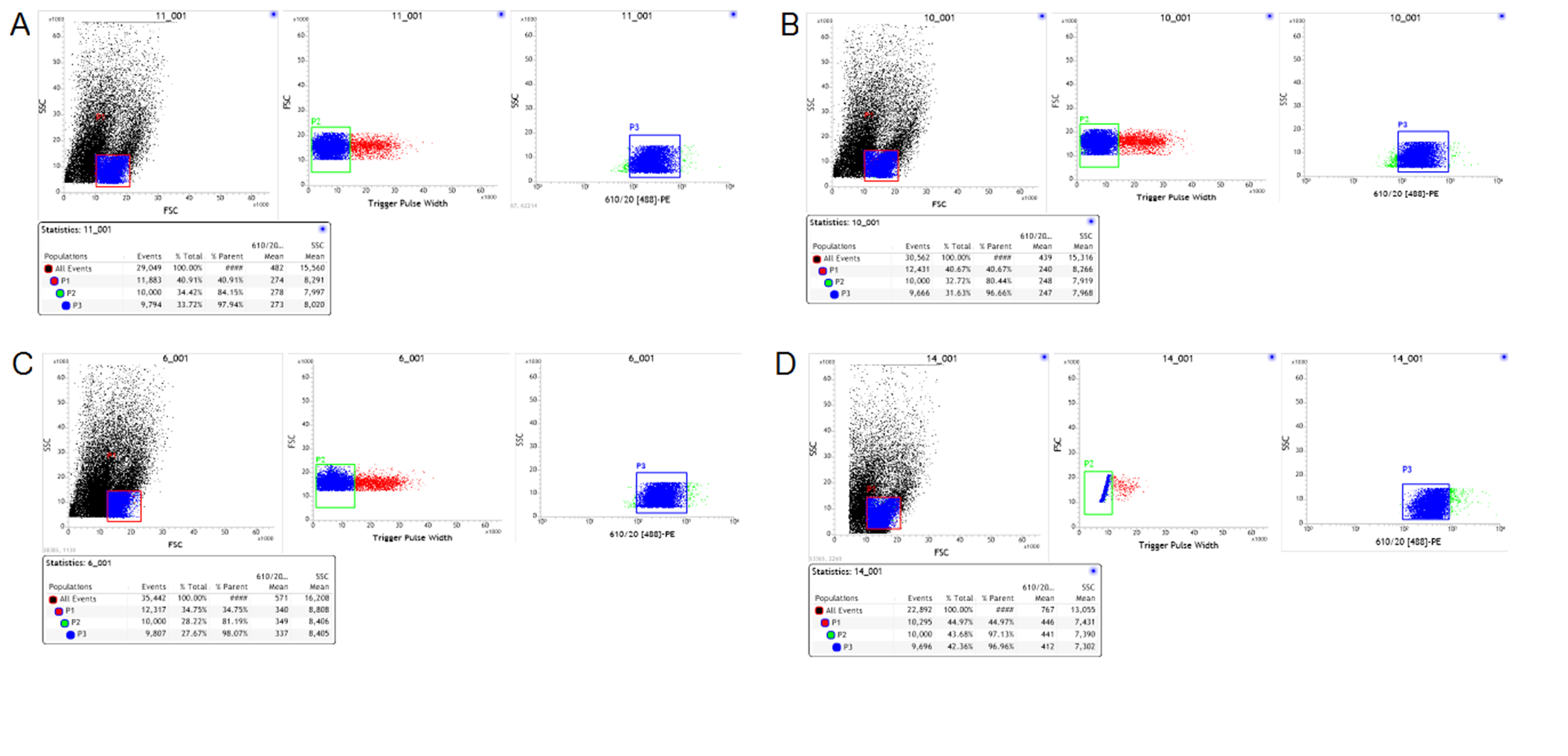

Supplement: Supplemental Information 3 — (A–D) Representative data from a multicolor assay of freshly isolated DC cells stained for PE derived from control rats, Model-control, RNAi-control and PDIA3-RNAi. Green-colored subpopulation indicates CD8+ cell; each number indicates the percentage in the parent population. [file peerj-04-2644-s006.png]

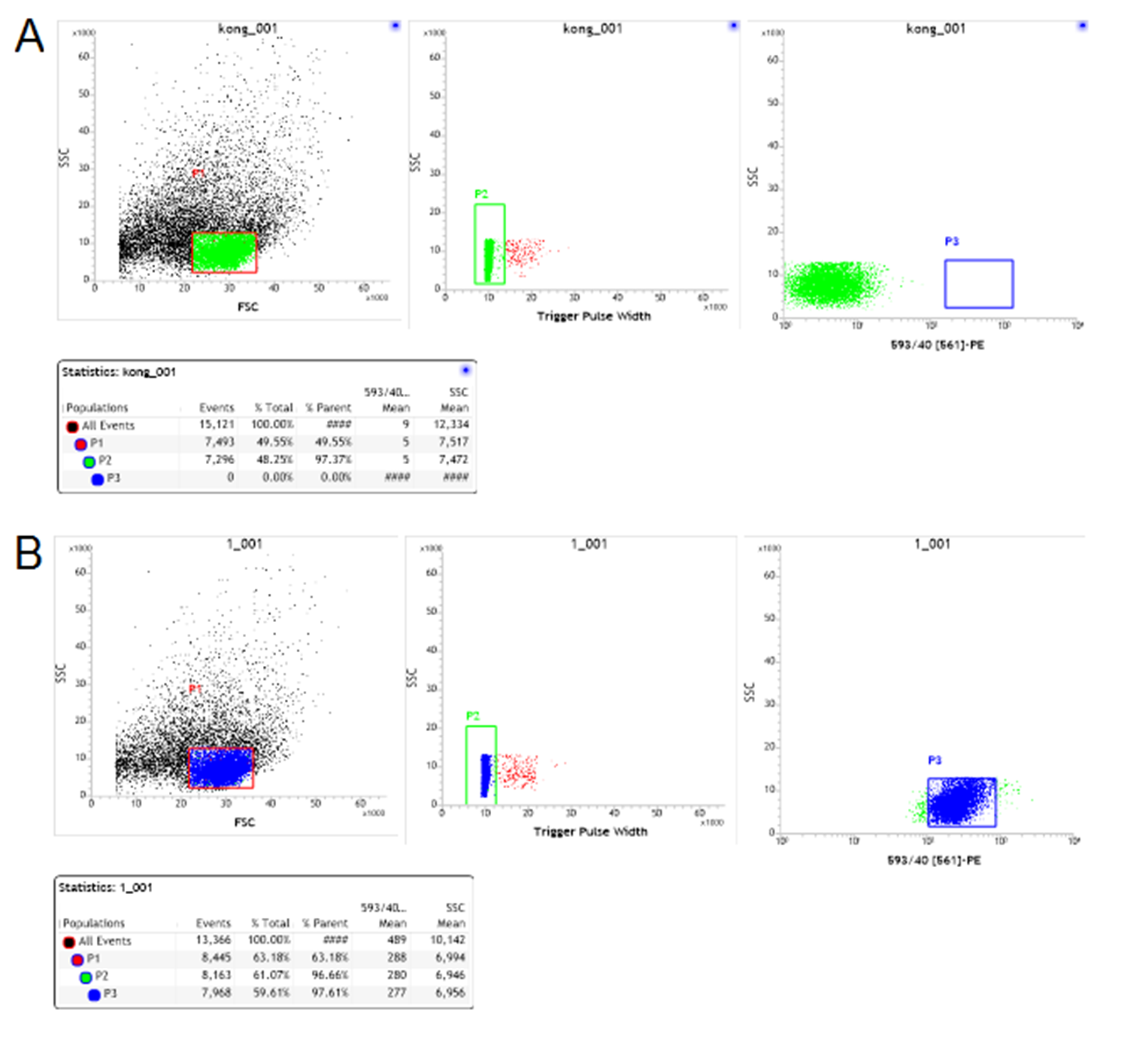

Supplement: Supplemental Information 4 — (A) and (B) Representative data from a multicolor assay of DC cells stained for CD80 after three days of culture without (A) and with (B) T cells. Blue-colored subpopulation indicates CD80+ cell; each number indicates the percentage in the parent population. [file peerj-04-2644-s007.png]
